# Solvent for Rubber

**Published:** 1877-06

**Authors:** 


*Solvent for Rubber.*—The *British Journal of Photography* describes a new solvent which consists of a mixture of methylated ether and petroleum spirits—the common benzolene used for burning in sponge lamps. This forms the most rapid and, perhaps, the best solvent we have tried ; the mixture is as much superior in power to either of its constituents singly as the ether-alcohol is to plain ether in its action on paroxylin. We make a very thick solution by dissolving sixty grains of good india rubber in two ounces of benzolene and one ounce of sulphuric ether. If the india rubber be cut up fine and the mixture shaken occasionally, the solution will be complete in two or three hours, when it may be diluted to any required strength with benzolene alone. The india rubber should be as light colored as possible, and all the outer oxidized portions must be cut away. Shred the clean india rubber with a pair of scissors, and throw it at once into the solvent.

